# Distinct Roles of Positive and Negative Maternal Mental Health in Parenting Styles and Child Development

**DOI:** 10.1016/j.jaacop.2025.11.007

**Published:** 2025-11-26

**Authors:** Michelle Z.L. Kee, Desiree Y. Phua, Helen Y. Chen, Yap Seng Chong, Lourdes Mary Daniel, Peter D. Gluckman, Birit F.P. Broekman, Michael J. Meaney

**Affiliations:** a Institute for Human Development and Potential, Agency for Science, Technology and Research (A∗STAR), Singapore, Republic of Singapore; b KK Women’s and Children’s Hospital, Singapore, Republic of Singapore; c Duke-NUS Medical School, Singapore, Republic of Singapore; d Yong Loo Lin School of Medicine, National University of Singapore, Singapore, Republic of Singapore; e Koi Tū Centre for Informed Futures, New Zealand; f Amsterdam UMC, Vrije Universiteit, Amsterdam, the Netherlands; g OLVG, Amsterdam, the Netherlands; h Amsterdam Mental Health, Amsterdam, the Netherlands; i Douglas Mental Health University Institute, McGill University, Montreal, Canada

**Keywords:** cognition, executive function, mental health, parenting, problem behavior

## Abstract

**Objective:**

Maternal mental health significantly impacts child development, yet the role of positive maternal mental health in shaping parenting and child outcomes remains unclear. This study examined how parenting styles mediate the relation between maternal mental health and developmental outcomes in 4- to 4.5-year-old children.

**Method:**

This study used data from the multiethnic Growing Up in Singapore Towards healthy Outcomes (GUSTO) cohort. A total of 328 mothers, who were recruited from Singapore hospital maternity units between 2009 and 2010, had completed longitudinal postnatal assessments of their mental health and parenting and child measures in English. Maternal mental health and parenting styles were assessed using the Beck Depression Inventory-II, State-Trait Anxiety Inventory, and Parenting Styles and Dimensions Questionnaire. Behavioral problems, school readiness, executive function, and IQ were assessed in children at 4 to 4.5 years old using validated behavioral and cognitive assessments.

**Results:**

Bifactor modeling revealed 2 distinct maternal mental health factors, general affective symptoms and positive mental health. The general affective symptoms factor was positively associated with permissive and authoritarian parenting (*r* = 0.27 and 0.29, *p*s < .001), which mediated the relation to child behavioral problems. Positive mental health correlated only with authoritative parenting (*r* = 0.19, *p* < .001), which mediated improvements in child executive function, verbal and numeracy abilities, and IQ.

**Conclusion:**

These findings highlight the distinct role of positive maternal mental health in fostering authoritative parenting, which in turn supports child cognitive development. Public health initiatives should prioritize promoting positive maternal mental health to enhance effective parenting and optimize child cognitive outcomes. Future research should consider paternal influences.

**Diversity & Inclusion Statement:**

We worked to ensure sex and gender balance in the recruitment of human participants. We worked to ensure race, ethnic, and/or other types of diversity in the recruitment of human participants. We worked to ensure that the study questionnaires were prepared in an inclusive way. Diverse cell lines and/or genomic datasets were not available. One or more of the authors of this paper self-identifies as a member of one or more historically underrepresented racial and/or ethnic groups in science. We actively worked to promote sex and gender balance in our author group.

## Plain language summary

A mother’s mental health shapes her child’s development, but less is known about the benefits of positive mental health. The multiethnic Growing Up in Singapore healthy Outcomes (GUSTO) longitudinal cohort study examined 328 mother-child pairs at baseline and at age 4-4.5 years old, including questionnaires, a home-based visit, and a laboratory-based visit. This study found that positive maternal mental health was linked to authoritative parenting, which in turn was linked to stronger cognitive abilities in children. Findings suggest that supporting mothers’ positive mental health can encourage supportive parenting styles and optimize cognitive outcomes in their children.

Maternal mental well-being affects parenting and child development.^1^ Although child maltreatment mediates the association between maternal depression and offspring risk for psychopathology,^2^ the link between maternal mood and variations in parenting is less compelling. Some, but not all, studies report anxious mothers to be less warm.^3^, ^4^, ^5^ The findings for depressed mothers are more consistent and stronger in mothers of low socioeconomic status.^6^^,^^7^ Maternal depression is associated with disengaged and hostile parenting; reduced sensitivity; and reduced positive affective behaviors, such as giving praise and constructive guidance.^7^, ^8^, ^9^ These parenting styles are associated with maternal depressive symptoms in both clinical and community samples, albeit with small effect sizes,^7^ underscoring the need to more comprehensively define the relation between maternal mood and parenting.^7^^,^^10^

Studies of maternal mental health and parenting are largely limited to symptoms of psychopathology, especially depression or anxiety. However, the quality of mental health is multidimensional and includes positive states such as optimism, self-confidence, and calmness.^11^ The World Health Organization noted that mental health quality cannot be defined simply by the absence of psychopathology.^12^ Negative and positive mental health are independent constructs^13^ with distinct antecedents,^14^ biological correlates,^15^ and unique contributions to health and mortality.^16^

Self-report screening tools for psychopathology, such as the General Health Questionnaire, provide measures of positive mental well-being.^14^ Phua *et al.*^17^ reported a bifactor modeling analysis of maternal self-report symptom scales that revealed a positive mental health factor and a general affective factor, which reflects symptom levels of both depression and anxiety. The positive mental health factor from this analysis is a distinct measure, only moderately anticorrelated with the general affective factor. The general affective factor is significantly associated with poorer socioemotional outcomes in children, whereas the positive mental health factor is more strongly associated with social, cognitive, and communication outcomes, suggesting a unique, outcome-specific influence of positive maternal mental health.^17^ These findings suggest a distinct pattern of association between positive mental health and general affective factors and specific child outcomes, underscoring the independence of these factors.

An obvious question is how positive maternal mental health influences child outcomes compared with maternal depression and anxiety. Whereas maternal depression and anxiety are well known to be associated with poor offspring outcomes, including impairments in socioemotional, language, and neurodevelopmental domains,^18^, ^19^, ^20^, ^21^ emerging evidence suggests that positive maternal mental health may exert protective effects.^21^ For example, positive maternal mental health is also associated with bilateral hippocampal enlargement in newborn girls,^22^ increased functional connectivity in offspring across several key neural networks such as thalamohippocampal and attention systems,^22^ and a lower risk of offspring developing mental and behavioral problems.^23^^,^^24^ Prior studies propose that disturbances in both negative and positive affect may be related to parenting, with reduced positive affect leading to less engagement during interactions.^7^^,^^25^ However, the relation between positive maternal mental health, parenting, and child outcomes remains unknown. Understanding this relation is pivotal for developing effective maternal mental health and parenting interventions that improve child outcomes.

To our knowledge, this is the first study to directly compare the associations of negative and positive maternal mental health and parenting style and to examine whether maternal parenting mediates the association between maternal mental health and child neurodevelopmental outcomes. We assessed parenting styles,^26^ including authoritarian, which values obedience and favors punitive disciplinary actions; permissive, which is nonpunitive and indulgent; and authoritative, which is warm, democratic, and rational. Child school readiness, executive function, IQ, and socioemotional problems were examined using both maternal reports and observational measures at ages 4 to 4.5. This approach allowed a definition of domain-specific pathways linking maternal mental health and parenting to child development. We hypothesized that positive maternal mental health is associated with authoritative parenting, which consequently mediates the associations between maternal mental health and specific child outcomes.

## Method

### Study Design and Participants

Healthy pregnant women (N = 1,257) ages ≥18 years were recruited into the Growing Up in Singapore Towards healthy Outcomes (GUSTO) longitudinal cohort study from hospital maternity units in Singapore between 2009 and 2010.^27^ Of these women, 1,049 participants remained in the cohort 4 years postnatally. However, due to limitations in study funding, personnel resources, and the logistical demands of the laboratory-based assessments, only 487 nontwin mother–child dyads were invited and consented to participate in postnatal longitudinal assessments of mood, parenting behaviors, and child measures, including a home-based visit and a laboratory-based visit when the child was 4 to 4.5 years old. Mothers (n = 328) who had completed all the maternal mental health–related questionnaires and the Parenting Styles and Dimensions Questionnaire (PSDQ) in English were included in the analyses. Maternal age and child gestational age at delivery were obtained from hospital records. Sociodemographic variables, including marital status, monthly household income, and maternal highest education level attained, were obtained during pregnancy. Informed consent was obtained from all participants. The study was approved by the National Health Care Group Domain Specific Review Board (D/09/02) and SingHealth Centralized Institutional Review Board (2009/280/D).

### Measures

#### Maternal Mental Health

Maternal depressive and anxiety symptoms were assessed using the Beck Depression Inventory–II (BDI-II)^28^ and State-Trait Anxiety Inventory (STAI),^29^ respectively, when the child was 4 years old. Only mothers who completed these questionnaires in English were assessed to ensure linguistic and semantic consistency among the items, which is essential for the validity of bifactor modeling.

#### Parenting Styles and Behaviors

Mothers completed the 32-item short version of the PSDQ^30^ when the child was 4.5 years old to report on parenting styles. Individual parenting style scores were obtained by averaging relevant parenting dimensions, as follows: warmth and supportive, reasoning and inductive, and democratic participative behavioral dimensions for authoritative parenting (α = .85); physically coercive, verbally hostile, and punitive dimensions for authoritarian parenting (α = .80); indulgent behavioral dimension for permissive parenting (α = .54) (see Table S1, available online, contains descriptive statistics). A multivariate analysis of variance revealed no significant associations (*p* > .05) between parenting styles and the child’s sex.

#### Child Outcomes

Socioemotional behavioral problems were assessed in the child at 4 years using the sum of 99 items from the maternal-reported Child Behavioral Checklist for Ages 1^1/2^-5 (CBCL/1^1/2^-5)^31^ (α = .95). During a home assessment, trained staff administered the Lollipop Test,^32^ the Number Knowledge Test (NKT),^33^ and the Peabody Picture Vocabulary Test Fourth Edition (PPVT-4).^34^ The Lollipop Test screens for school readiness. The NKT assesses number knowledge using whole numbers up to 10. The PPVT-4 evaluates receptive vocabulary. Total raw scores were used for the Lollipop Test, NKT, and PPVT-4, with higher scores reflecting better performance. Executive function was measured using the Cambridge Neuropsychological Test Automated Battery (CANTAB) Spatial Working Memory^35^ task when the child was 4.5 years old. The task assesses spatial working memory and heuristic strategies by having children locate a blue token among 3 to 8 boxes. Total errors for 4- to 8-box experiments served as the outcome measure. Revisiting empty boxes or those with previously found tokens increases total errors, indicating poorer spatial working memory. The Kaufman Brief Intelligence Test Second Edition (KBIT-2)^36^ measured global intelligence at 4.5 years. The composite IQ score, derived from verbal and nonverbal sections and standardized with a mean (SD) score of 100 (15), was used. Descriptive statistics for all outcomes are presented in Table S1, available online.

### Statistical Analyses

#### Bifactor Models

Bifactor latent class was used to define dimensions of maternal mental health as previously described.^17^ The BDI-II and STAI items were fitted to exploratory bifactor models with bi-geomin oblique and orthogonal rotations, with 1,000 iterations and a maximum likelihood robust estimator. Model fit was assessed by comparing eigenvalues and fit indices of the unidimensional (1-factor) model with those of the bifactor model. Fit indices include Akaike information criterion, Bayesian information criterion, root mean square error of approximation (RMSEA), comparative fit index (CFI), and standardized root mean squared residual (SRMR). The number of factors was determined by parallel analysis, retaining factors with eigenvalues higher than the corresponding randomly generated eigenvalue.^37^ The best-fitting exploratory model with loadings ≥ |0.30| was reestimated as a confirmatory model to obtain factor scores for subsequent analyses. The confirmatory model fit was evaluated using CFI and Tucker-Lewis index >0.90, SRMR <0.07, RMSEA <0.05, and the model χ^2^ test.^38^ We assessed the reliability of the factors from the bifactor confirmatory model using explained common variance, ω coefficient for internal reliability (acceptable if >0.7),^39^ ω h (percentage of raw score variance attributed to individual differences in the factor), relative ω (the ratio of ω to ωh, representing the proportion of reliable variance),^40^ *H* index for construct reliability (minimum threshold of 0.80),^41^ and factor determinacy index (recommended threshold of 0.90).^42^

#### Correlation and Mediation Analyses

Spearman ρ analyses between maternal mental health, parenting styles, and child outcomes were performed using Hmisc::rcorr function in R.^43^ Mediation analyses were conducted in Mplus^44^ to examine whether parenting styles mediated the associations between maternal well-being and child outcomes. To maximize participant data without introducing imputing bias, each mediation model included maternal positive mental health or general affective symptoms as the independent variable; each specific child outcome as the dependent variable; and permissive, authoritarian, or authoritative parenting styles as mediators. Direct effect is defined as the effect of the causal pathway between the independent variable (maternal mental health) and the dependent variable (child outcomes) in the absence of the mediator (parenting style). Total effect is defined as the sum of the direct and indirect (mediational) effects. Mediation effects were based on 5,000 bias-corrected (BC) bootstrap samples with a 95% CI and repeated with a 99% CI for a more conservative estimate of the effect. Significant mediation was determined when the bootstrap CIs for the indirect effect did not contain zero value, even when the total or direct effects are nonsignificant.^45^^,^^46^ Maternal ethnicity and the highest education level attained were added as covariates to the mediation models as they were significantly associated with certain parenting styles and child outcomes. Specifically, maternal ethnicity was associated with authoritative parenting and child school readiness, number knowledge, receptive vocabulary, IQ, and total errors in spatial working memory, but not with child behavioral problems. Similarly, maternal education was associated with all child outcomes except for behavioral problems. Accordingly, these covariates were included in the relevant mediation models. Multiple testing corrections were performed using the Benjamini-Hochberg correction with a false discovery rate *p*-value threshold of .05 to control for type I errors. Adjusted *p* values < .05 are considered significant. Unadjusted models are presented in Tables S8 and S9, available online.

## Results

### Study Characteristics

Table 1 summarizes the study demographics. The 328 mothers who participated in the study were mostly Chinese (169 [51.5%]), reflecting the Singaporean population distribution, and had a mean (SD) age of 31 (5) years at delivery, with approximately half delivering a boy (168 [51.2%]). Children were born full-term at a mean (SD) of 38.76 (1.39) gestational weeks. Most mothers attained at least pretertiary education levels (248 [76.5%]) and were married (312 [97.5%]) at delivery. Mothers in our study did not differ in demographic characteristics from mothers in the larger GUSTO cohort (*p*s > .05) (Table 1). Also, no differences in demographic characteristics were observed between participants who were invited and consented to longitudinal postnatal assessments and the participants included in our study except for the highest education level attained by the mothers (χ^2^_2,806_ = 7.04, *p* = .03) (Table S2, available online). Pairwise comparisons showed that mothers in our study were more likely to attain a university degree as opposed to secondary education or below (Holm-adjusted *p* = .033). No significant differences were observed between other educational groups (all *p*s > .05).

**Table 1:** Demographic Characteristics of Participants in Larger GUSTO Cohort and Current Study

	GUSTO cohort (N = 1,049)	Current study (n = 328)			*p*^a^
	**Mean**	**(SD)**	**Mean**	**(SD)**	
Mother’s age at delivery, y	31	(5)	31	(5)	>.900
	**n**	**(%)**	**n**	**(%)**	
Ethnicity					.100
Chinese	591	(56.3)	169	(51.5)	
Indian	192	(18.3)	56	(17.1)	
Malay	266	(25.4)	103	(31.4)	
Maternal highest education level					.094^b^
Secondary and below	315	(30.0)	76	(23.5)	
Pretertiary	361	(34.4)	117	(36.1)	
University and above	361	(34.4)	131	(40.4)	
Missing data/refused to answer	12	(1.1)	4	(1.2)	
Monthly household income (SGD)^c^					.208
≤1,999	148	(14.1)	33	(10.0)	
2,000-3,999	299	(28.5)	89	(27.1)	
4,000-5,999	241	(23.0)	79	(24.1)	
>6,000	292	(27.8)	108	(32.9)	
Missing data/refused to answer	69	(6.6)	19	(5.8)	
Marital status					.681
Married	996	(94.9)	312	(95.1)	
Single	33	(3.1)	8	(2.4)	
Missing/refused to answer	20	(1.9)	8	(2.4)	
Child sex, male	553	(52.7)	168	(51.2)	.700
	**Mean**	**(SD)**	**Mean**	**(SD)**	
Child gestational age at delivery, wk	38.77	(1.47)	38.76	(1.39)	>.900
Maternal measures					
BDI-II	6.24	(7.36)	6.24	(7.56)	>.900
	**n**	**(%)**	**n**	**(%)**	
Missing	619	(59.0)	—		
	**Mean**	**(SD)**	**Mean**	**(SD)**	
STAI-State	33.85	10.26	33.30	10.58	.473
	**n**	**(%)**	**n**	**(%)**	
Missing	620	(59.1)	—		
	**Mean**	**(SD)**	**Mean**	**(SD)**	
STAI-Trait	36.36	9.67	36.05	10.01	.664
	**n**	**(%)**	**n**	**(%)**	
Missing	620	(59.1)	—		

Note: BDI-II = Beck Depression Inventory–II; GUSTO = Growing Up in Singapore Towards healthy Outcomes; SGD = Singapore dollar; STAI = State-Trait Anxiety Inventory.

a Welch 2-sample t test for continuous variables and Pearson’s χ^2^ test for categorical variables.

b Fisher’s exact test.

c The acknowledged poverty line is SGD $1,999 monthly income.

### Distinct Factors of Maternal Mental Health

Mothers had mean (SD) BDI-II, STAI State, and STAI Trait scores of 6.24 (7.56), 33.30 (10.58), and 36.05 (10.01), respectively. BDI-II and STAI items were analyzed in an exploratory bifactor model to uncover positive and negative dimensions of mental health not captured by total scale scores. Oblique and orthogonal bifactor models were tested, but the oblique model failed to converge. The orthogonal bifactor models yielded significant loadings for these individual factors of mental health, indicating that domain-specific variance was captured in addition to the general factor. The eigenvalues and fit indices (Akaike information criterion, Bayesian information criterion, RMSEA, CFI, and SRMR) of the unidimensional (1-factor) model were compared with other factors from the exploratory bifactor modeling (Table S3, available online). The bifactor models consistently showed improved fit across multiple indices relative to that of the unidimensional model (eg, ΔCFI = +0.262; RMSEA decreased from 0.093 to 0.056).

The eigenvalues of the 5-factor model, but not the 6-factor model, exceeded the corresponding randomly generated eigenvalues. Hence, the 5-factor model was subsequently reestimated as the confirmatory model. This 5-factor model consisted of a general factor and 4 specific subfactors—positive mental health, sadness, psychosomatic, and anxiety (Tables S4 and S5, available online). The confirmatory model had reasonably good fit (χ^2^_1727,328_ = 2,900.48, *p* < .001; RMSEA = 0.05, *p* < .001; CFI = 0.96; Tucker-Lewis index = 0.96; SRMR = 0.06). The general factor was interpreted as a general affective symptoms factor reflecting an integration of anxiety and depression symptoms and compared with the positive mental health subfactor in subsequent analyses. These 2 factors showed the highest reliability on all the indices and the greatest explained variance in the confirmatory model (73.7% and 42.8% for general affective symptoms and positive mental health factors, respectively). Both general affective symptoms and positive mental health factors had high internal reliability and construct replicability (Table S6, available online), suggesting that the 2 latent factors were well defined. A higher score on the general affective symptoms factor reflects a more negative affective state (ie, symptoms of depression and anxiety). A higher score on the positive mental health factor independently reflects a more positive affective state (eg, feeling satisfied). The general affective symptoms factor was also anticorrelated with positive mental health factor scores (*r* = −0.12; *p* = .034) (Table 2).

**Table 2:** Correlations Between Factor Scores of Maternal General Affective Symptoms, Positive Mental Health, Parenting Styles, and Child Outcomes

	1	2	3	4	5	6	7	8	9	10	11
1. General affective symptoms	—	—	—	—	—	—	—	—	—	—	—
2. Positive mental health	−0.12 (.034)	—	—	—	—	—	—	—	—	—	—
3. Authoritative parenting	−0.18 (.001)	0.19 (.001)	—	—	—	—	—	—	—	—	—
4. Authoritarian parenting	0.29 (<.001)	−0.01 (.810)	−0.14 (.011)	—	—	—	—	—	—	—	—
5. Permissive parenting	0.27 (<.001)	0.01 (.831)	−0.06 (.278)	0.43 (<.001)	—	—	—	—	—	—	—
6. School readiness	−0.002 (.966)	0.09 (.122)	0.09 (.110)	0.01 (.890)	0.09 (.101)	—	—	—	—	—	—
7. Number knowledge	0.03 (.648)	0.08 (.157)	0.11 (.066)	−0.05 (.426)	−0.001 (.982)	0.63 (<.001)	—	—	—	—	—
8. Receptive vocabulary	−0.10 (.097)	0.15 (.009)	0.12 (.034)	−0.02 (.753)	0.07 (.255)	0.63 (<.001)	0.48 (<.001)	—	—	—	—
9. IQ	−0.12 (.037)	0.20 (.001)	0.11 (.069)	−0.17 (.003)	0.01 (.893)	0.52 (<.001)	0.47 (<.001)	0.68 (<.001)	—	—	—
10. Total errors in spatial working memory	−0.003 (.954)	−0.03 (.655)	−0.06 (.295)	0.03 (.628)	−0.04 (.462)	−0.26 (<.001)	−0.24 (<.001)	−0.15 (.017)	−0.19 (.002)	—	—
11. Total behavioral problems	0.42 (<.001)	0.004 (.941)	−0.11 (.071)	0.26 (<.001)	0.26 (<.001)	−0.05 (.371)	−0.05 (.417)	−0.10 (.097)	−0.08 (.222)	−0.02 (.688)	—

Note: *p* values are within parentheses; n = 266–328.

### Associations Between Maternal Mental Health Factors, Parenting Styles, and Child Outcomes

We examined the associations between maternal mental health factors and parenting styles (Table 2) and specific parenting dimensions (Table S7, available online). The general affective symptoms factor correlated positively with authoritarian and permissive parenting (*r* = 0.29 and 0.27, respectively, *p*s < .001). This factor also correlated positively with physically coercive (*r* = 0.20, *p* < .001), verbally hostile (*r* = 0.24, *p* < .001), and nonreasoning and punitive parenting behaviors (*r* = 0.24, *p* < .001). Conversely, the general affective symptoms factor was anticorrelated with authoritative parenting (*r* = −0.18, *p* = .001) and related parenting behaviors, specifically warmth and supportive (*r* = −0.16, *p* = .003), reasoning and inductive, and democratic participative parenting behaviors (both *r* = −0.16, *p*s = .004). Conversely, the maternal positive mental health factor was positively associated with authoritative parenting (*r* = 0.19, *p* = .001) and related parenting behaviors (warmth and supportive: *r* = 0.14, *p* = .012; reasoning and inductive: *r* = 0.23, *p* < .001; and democratic participative: *r* = 0.12, *p* = .027). Positive mental health was not associated with permissive and authoritarian parenting, reflecting the independence of the 2 maternal well-being factors.

Maternal general affective symptoms factor was also negatively associated with child IQ (*r* = −0.12, *p* = .037) and positively associated with child behavioral problems (*r* = 0.42, *p* < .001). However, maternal positive mental health showed positive associations with child receptive vocabulary (*r* = 0.15, *p* = .009) and IQ (*r* = 0.20, *p* = .001).

We next examined associations of parenting styles and dimensions with the child outcomes. Authoritative parenting was positively associated with child receptive vocabulary (*r* = 0.12, *p* = .034). This is primarily driven by reasoning and inductive parenting behavior (*r* = 0.20, *p* < .001). Children whose mothers reported higher reasoning and inductive parenting behaviors also had stronger numeracy ability (*r* = 0.19, *p* = .001) and higher IQ (*r* = 0.16, *p* = .005). In contrast, warmth and supportive and democratic participative authoritative-related behaviors were not significantly correlated with child number knowledge, receptive vocabulary, or IQ. Authoritarian parenting was associated with lower child IQ (*r* = −0.17, *p* = .003), with similar negative associations observed for physical coercion (*r* = −0.17, *p* = .003) and nonreasoning behaviors (*r* = −0.16, *p* = .007). Maternal nonreasoning behavior was also associated with lower child number knowledge (*r* = −0.14, *p* = .017). Child behavioral problems were positively associated with permissive and authoritarian parenting (both *r* = 0.26, *p* < .001) and authoritarian-related parenting behaviors (physical coercion: *r* = 0.18, *p* = .002; verbal hostility: *r* = 0.24, *p* < .001; nonreasoning: *r* = 0.19, *p* = .001). Authoritative parenting, however, was not associated with child behavioral problems. No association was observed between maternal mental health, parenting styles, and specific parenting behaviors with school readiness or executive function.

### Mediation Effects of Parenting Styles on Relation Between Maternal General Affective Symptoms and Child Outcomes

We assessed the specific parenting styles as mediators between maternal mental health and child outcomes (Tables 3 and 4) by using a 95% CI BC bootstrapping method with covariates added to the relevant mediation models (see “Method” and Table S8, available online). Maternal general affective symptoms were consistently associated with higher levels of child behavioral problems across all parenting styles (total effects: β_authoritarian_ [95% CI] = .44 [.35, .53]; β_authoritative_ [95% CI] = .44 [.35, .53]; β_permissive_ [95% CI] = .44 [.35, .53]). These associations remained significant after adding parenting as mediators, showing the direct influences of maternal general affective symptoms on child behavioral problems (β_authoritarian_ [95% CI] = .41 [.30, .50]; β_authoritative_ [95% CI] = .44 [.34, .53]; β_permissive_ [95% CI] = .40 [.30, .50]) (Table 3). Additionally, significant indirect effects were observed through authoritarian (β [95% CI] = .04 [.004, .083], *p*_adj_ = .023) and permissive parenting (β [95% CI] = .04 [.01, .08], *p*_adj_ = .005). Conversely, authoritative parenting did not mediate the relation between maternal general affective symptoms and child behavioral problems. To further assess the robustness of the mediation pathway, we conducted BC bootstrap mediation analyses using a more conservative 99% CI. Permissive parenting remained a significant mediator between maternal general affective symptoms and child socioemotional problems (β [99% CI] = .04 [.003, .095], *p*_adj_ = .005), reinforcing our earlier findings from the 95% CI. These findings suggest that permissive and authoritarian parenting were the only mediators between elevated general affective symptoms and increased child socioemotional problems.

**Table 3:** Adjusted Mediation Effects of Parenting Styles on Relation Between Maternal General Affective Symptoms and Various Child Outcomes

Child outcomes	n	Model	Effect type	Mediator: authoritarian^a^				Mediator: authoritative^b^				Mediator: permissive^a^			
				β	[95% CI]	β	[99% CI]	β	[95% CI]	β	[99% CI]	β	[95% CI]	β	[99% CI]
School readiness	297	Adjusted	Total	.016	[−0.095, 0.120]	.016	[−0.131, 0.153]	.014	[−0.097, 0.118]	.014	[−0.135, 0.153]	.017	[−0.094, 0.120]	.017	[−0.131, 0.154]
			Direct	.018	[−0.103, 0.129]	.018	[−0.144, 0.168]	.037	[−0.075, 0.147]	.037	[−0.114, 0.176]	.013	[−0.103, 0.121]	.013	[−0.138, 0.160]
			Indirect	−.001	[−0.034, 0.032]	−.001	[−0.045, 0.044]	**−.023**	**[−0.057, −0.003]**∗	−.023	[−0.072, 0.003]	.004	[−0.029, 0.036]	.004	[−0.042, 0.048]
Number knowledge	288	Adjusted	Total	.051	[−0.066, 0.155]	.051	[−0.096, 0.189]	.046	[−0.070, 0.156]	.046	[−0.104, 0.189]	.052	[−0.065, 0.156]	.052	[−0.096, 0.19]
			Direct	.078	[−0.043, 0.195]	.078	[−0.074, 0.227]	.070	[−0.047, 0.183]	.070	[−0.083, 0.215]	.061	[−0.065, 0.180]	.061	[−0.100, 0.213]
			Indirect	−.028	[−0.067, 0.003]	−.028	[−0.080, 0.0130]	**−.024**	**[−0.054, −0.004]**	−.024	[−0.064, 0.001]	−.009	[−0.045, 0.025]	−.009	[−0.060, 0.035]
Receptive vocabulary	289	Adjusted	Total	−.049	[−0.165, 0.069]	−.049	[−0.206, 0.098]	−.053	[−0.172, 0.066]	−.053	[−0.214, 0.098]	−.049	[−0.164, 0.069]	−.049	[−0.205, 0.098]
			Direct	−.038	[−0.154, 0.077]	−.038	[−0.191, 0.112]	−.025	[−0.144, 0.098]	−.025	[−0.187, 0.136]	−.049	[−0.168, 0.072]	−.049	[−0.203, 0.110]
			Indirect	−.011	[−0.041, 0.018]	−.011	[−0.052, 0.030]	**−.029**	**[−0.059, −0.009]**∗∗∗	**−.029**	**[−0.072,−0.004]**∗∗∗	0	[−0.031, 0.030]	0	[−0.043, 0.043]
IQ	296	Adjusted	Total	−.060	[−0.164, 0.052]	−.060	[−0.196, 0.086]	−.059	[−0.168, 0.054]	−.059	[−0.203, 0.095]	−.060	[−0.165, 0.051]	−.060	[−0.197, 0.086]
			Direct	−.023	[−0.135, 0.089]	−.023	[−0.166, 0.131]	−.034	[−0.146, 0.083]	−.034	[−0.183, 0.120]	−.060	[−0.178, 0.056]	−.060	[−0.21, 0.093]
			Indirect	**−.037**	**[−0.075, −0.008]**∗∗∗	**−.037**	**[−0.089, −0.001]**∗∗∗	**−.025**	**[−0.055, −0.005]**∗	−.025	[−0.067, 0.001]	.001	[−0.031, 0.030]	.001	[−0.044, 0.040]
Total errors in spatial working memory^c^	296	Adjusted	Total	.006	[−0.111, 0.121]	.006	[−0.147, 0.155]	.021	[−0.092, 0.127]	.021	[−0.129, 0.164]	.007	[−0.111, 0.120]	.007	[−0.146, 0.156]
			Direct	−.010	[−0.135, 0.109]	−.010	[−0.172, 0.153]	−.001	[−0.114, 0.109]	−.001	[−0.151, 0.145]	.012	[−0.111, 0.132]	.012	[−0.145, 0.176]
			Indirect	.016	[−0.015, 0.053]	.016	[−0.027, 0.065]	**.022**	**[0.004, 0.052]**∗	.022	[−0.002, 0.063]	−.005	[−0.043, 0.027]	−.005	[−0.057, 0.04]
															
Total behavioral problems^d^	289	Unadjusted	Total	.442	[0.349, 0.527]	.442	[0.318, 0.552]	.442	[0.352, 0.530]	.442	[0.319, 0.556]	.440	[0.347, 0.525]	.440	[0.315, 0.551]
			Direct	.405	[0.302, 0.502]	.405	[0.266, 0.531]	.437	[0.340, 0.527]	.437	[0.307, 0.556]	.402	[0.301, 0.495]	.402	[0.268, 0.522]
			Indirect	**.037**	**[0.004, 0.083]**∗	.037	[−0.006, 0.100]	.006	[−0.014, 0.032]	.006	[−0.023, 0.042]	**.037**	**[0.010, 0.081]**∗∗	**.037**	**[0.003, 0.095]**∗∗

Note: β = standardized estimates; 95% CI and 99% CI = bias-corrected bootstrapping estimation method with a 95% or 99% CI. Boldface values indicate significant indirect effects (ie, 95% or 99% CI do not cross zero).

a Adjusted models include maternal highest education level attained as covariates, except when the model includes total behavioral problems as outcome.

b Adjusted models include maternal ethnicity and maternal highest education level attained as covariates, except when the model includes total behavioral problems as outcome.

c From Cambridge Neuropsychological Test Automated Battery (CANTAB) Spatial Working Memory task.

d From Childhood Behavioral Checklist for Ages 1^1/2^-5 (CBCL/1^1/2^-5).

∗ *p* < .05; ∗∗*p* < .01; ∗∗∗*p* < .005 after multiple testing corrections.

**Table 4:** Adjusted Mediation Effects of Parenting Styles on Relation Between Maternal Positive Mental Health and Various Child Outcomes

Child outcomes	n	Models	Effect type	Mediator: authoritarian^a^				Mediator: authoritative^b^				Mediator: permissive^a^			
				β	[95% CI]	β	[99% CI]	β	[95% CI]	β	[99% CI]	β	[95% CI]	β	[99% CI]
School readiness	297	Adjusted	Total	.068	[−0.033, 0.174]	.068	[−0.068, 0.204]	.066	[−0.033, 0.172]	.066	[−0.072, 0.202]	.068	[−0.031, 0.176]	.068	[−0.067, 0.204]
			Direct	.068	[−0.033, 0.175]	.068	[−0.067, 0.203]	.043	[−0.059, 0.153]	.043	[−0.095, 0.191]	.067	[−0.033, 0.174]	.067	[−0.067, 0.204]
			Indirect	0	[−0.006, 0.008]	0	[−0.011, 0.013]	.023	[0, 0.059]	.023	[−0.008, 0.074]	.001	[−0.004, 0.012]	.001	[−0.007, 0.018]
Number knowledge	288	Adjusted	Total	.037	[−0.080, 0.158]	.037	[−0.115, 0.191]	.040	[−0.074, 0.156]	.040	[−0.104, 0.191]	.037	[−0.08, 0.157]	.037	[−0.113, 0.191]
			Direct	.039	[−0.078, 0.158]	.039	[−0.111, 0.189]	.017	[−0.099, 0.136]	.017	[−0.130, 0.172]	.037	[−0.08, 0.158]	.037	[−0.113, 0.192]
			Indirect	−.002	[−0.019, 0.006]	−.002	[−0.027, 0.010]	**.023**	**[0.001, 0.057]**∗	.023	[−0.005, 0.068]	−.001	[−0.012, 0.004]	−.001	[−0.018, 0.007]
Receptive vocabulary	289	Adjusted	Total	.094	[−0.019, 0.209]	.094	[−0.051, 0.247]	.103	[−0.009, 0.215]	.103	[−0.046, 0.252]	.095	[−0.019, 0.209]	.095	[−0.050, 0.247]
			Direct	.095	[−0.020, 0.209]	.095	[−0.050, 0.253]	.072	[−0.042, 0.185]	.072	[−0.074, 0.220]	.095	[−0.020, 0.211]	.095	[−0.051, 0.249]
			Indirect	−.001	[−0.015, 0.005]	−.001	[−0.023, 0.008]	**.031**	**[0.009, 0.066]**∗∗∗	**.031**	**[0.004, 0.081]**∗∗∗	−.001	[−0.013, 0.003]	−.001	[−0.019, 0.006]
IQ	296	Adjusted	Total	.134	[0.024, 0.244]	.134	[−0.009, 0.276]	.132	[0.023, 0.240]	.132	[−0.005, 0.274]	.131	[0.022, 0.242]	.131	[−0.011, 0.275]
			Direct	.137	[0.026, 0.246]	.137	[−0.006, 0.280]	.107	[−0.007, 0.229]	.107	[−0.040, 0.266]	.132	[0.022, 0.243]	.132	[−0.010, 0.275]
			Indirect	−.003	[−0.022, 0.013]	−.003	[−0.029, 0.019]	**.025**	**[0.003, 0.064]**∗	.025	[−0.005, 0.081]	−.001	[−0.012, 0.003]	−.001	[−0.015, 0.006]
Total errors in spatial working memory^a^^,^^c^	296	Adjusted	Total	−.019	[−0.129, 0.094]	−.019	[−0.160, 0.124]	−.028	[−0.135, 0.084]	−.028	[−0.164, 0.117]	−.018	[−0.128, 0.094]	−.018	[−0.160, 0.123]
			Direct	−.021	[−0.13, 0.092]	−.021	[−0.163, 0.123]	−.002	[−0.112, 0.113]	−.002	[−0.150, 0.144]	−.018	[−0.127, 0.096]	−.018	[−0.160, 0.128]
			Indirect	.001	[−0.005, 0.016]	.001	[−0.009, 0.022]	**−.026**	**[−0.061, −0.003]**∗∗	−.026	[−0.075, 0.005]	−.001	[−0.012, 0.004]	−.001	[−0.017, 0.008]
Total behavioral problems^b^^,^^d^	289	Unadjusted	Total	−.048	[−0.165, 0.072]	−.048	[−0.196, 0.106]	−.049	[−0.166, 0.071]	−.049	[−0.199, 0.105]	−.044	[−0.159, 0.076]	−.044	[−0.193, 0.112]
			Direct	−.051	[−0.167, 0.067]	−.051	[−0.201, 0.100]	−.026	[−0.143, 0.097]	−.026	[−0.173, 0.135]	−.051	[−0.162, 0.062]	−.051	[−0.199, 0.097]
			Indirect	.003	[−0.026, 0.032]	.003	[−0.038, 0.042]	−.023	[−0.063, 0.001]	−.023	[−0.076, 0.010]	.008	[−0.017, 0.037]	.008	[−0.029, 0.048]

Note: β = standardized estimates; 95% CI and 99% CI = bias-corrected bootstrapping estimation method with a 95% or 99% CI. Boldface values indicate significant indirect effects (ie, 95% or 99% CI do not cross zero).

a Adjusted models include maternal highest education level attained as covariates, except when the model includes total behavioral problems as outcome.

b Adjusted models include maternal ethnicity and maternal highest education level attained as covariates, except when the model includes total behavioral problems as outcome.

^c^ From Cambridge Neuropsychological Test Automated Battery (CANTAB) Spatial Working Memory task.

^d^ From Childhood Behavioral Checklist for Ages 1^1/2^-5 (CBCL/1^1/2^-5).

∗ *p* < .05; *∗∗p* < .01; ∗∗∗*p* < .005 after multiple testing corrections.

We next examined the relation between maternal general affective symptoms, parenting styles, and child IQ. Although no significant total or direct effects were observed, authoritative and authoritarian parenting significantly mediated the relation between maternal general affective symptoms and child IQ (β_authoritative_ [95% CI] = −.03 [−.055, −.005], *p*_adj_ = .011; β_authoritarian_ [95% CI] = −.04 [.075, −.008], *p*_adj_ = .003). Notably, mediation through authoritarian parenting also survived the more conservative 99% CI BC mediation analyses (β [95% CI] = −.037 [−.089, −.001], *p*_adj_ = .003), indicating a robust mediation effect.

We next repeated the analyses with child school readiness (including number knowledge and receptive vocabulary) and executive function as outcomes. Authoritative parenting significantly mediated the relation between maternal general affective symptoms and these child outcomes (total errors in spatial working memory: β [95% CI] = −.023 [−.057, −.003], *p*_adj_ = .020; school readiness: β [95% CI] = −0.023 [−0.057, −0.003], *p*_adj_ = .036; number knowledge: β [95% CI] = −0.024 [−0.054, −0.004], *p*_adj_ = .017; receptive vocabulary: β [95% CI] = −0.029 [−0.059, −0.009], *p*_adj_ = .002). Although no significant direct and total effects were observed, the indirect effects on school readiness, number knowledge, and executive function were in the opposite direction of the direct and total effects. Additionally, authoritative parenting remained a significant mediator between maternal general affective symptoms and child receptive vocabulary ability under the more stringent 99% CI BC approach (β [99% CI] = −.029 [−.072, −.004], *p*_adj_ = .002), indicating a robust indirect effect. Notably, after multiple testing corrections, authoritative parenting remained a significant mediator between general affective symptoms and school readiness, number knowledge, receptive vocabulary, and executive function. These findings underscore the critical and complex interplay between authoritative parenting, maternal general affective symptoms, and their influence on child’s preacademic skills and self-regulation abilities.

### Mediation Effects of Parenting Styles on Relation Between Maternal Positive Mental Health and Child Outcomes

Table 4 summarizes the associations between positive maternal mental health, parenting styles, and child outcomes, including the mediation effects of parenting styles (Table S9, available online, contains full detailed results). Positive maternal mental health remained significantly associated with higher child IQ (total effects: β [95% CI] = .13 [.02, .24] for authoritarian, authoritative, and permissive pathways). Positive maternal mental health remained directly associated with child IQ when authoritarian and permissive parenting were included as mediators (β_authoritarian_ [95% CI] = .14 [.03, .25]; β_permissive_ [95% CI] = .13 [.02, .24]), whereas the indirect effects were nonsignificant. This suggests that the positive association between maternal positive mental health and child IQ is primarily direct and not mediated by authoritarian or permissive parenting. Conversely, authoritative parenting significantly mediated the association between maternal positive mental health and child IQ (β [95% CI] = .025 [.003, .064], *p*_adj_ = .022), whereas the direct effect was nonsignificant, suggesting that the influence of positive maternal mental health on child IQ operates primarily through authoritative parenting.

No significant direct and total associations were observed between maternal positive mental health and child outcomes in school readiness, number knowledge, receptive vocabulary, and executive function. However, authoritative parenting significantly mediated the relation on child numeracy ability (β [95% CI] = .023 [.001, .057], *p*_adj_ = .017) and executive function, as reflected by fewer total errors in spatial working memory (β [95% CI] = −.026 [−.061, −.003], *p*_adj_ = .008). Authoritative parenting also showed a robust indirect effect between maternal positive mental health on child receptive vocabulary (β [95% CI] = .031 [.009, .066], *p*_adj_ = .002), even under the stringent 99% CI BC analyses (β [99% CI] = .031 [.004, .081], *p*_adj_ = .002). These findings support a mechanistic pathway, whereby authoritative parenting acts as a key conduit through which positive maternal mental health is associated with better executive function, numeracy, IQ, and especially receptive verbal performance in school readiness tests.

In contrast, permissive and authoritarian parenting did not mediate the relation between positive maternal mental health and child developmental outcomes. No parenting style mediated the association between positive maternal mental health and child socioemotional problems. These results highlight the pivotal role of authoritative parenting in mediating the relation of positive maternal mental health with specific influences on child cognitive outcomes.

## Discussion

Our primary objective was to comprehensively assess maternal mental health in both positive and negative dimensions and delineate specific associations with child outcomes, focusing on the mediating role of parenting style. Notably, our analyses uniquely prioritized positive maternal mental health. Our findings reveal significant relations between positive maternal mental health and child cognitive function, underscoring the interplay between positive maternal mental health and authoritative parenting in these associations.

Bifactor modeling with screening tools for depression (BDI-II) and anxiety (STAI) revealed 2 factors, general affective symptoms and positive mental health. The general affective symptoms factor represents a latent construct capturing the shared variance between maternal anxiety and depressive symptoms, reflecting their often co-occurring nature. Higher scores on this factor indicate greater severity on this shared dimension, but do not distinguish whether elevations are primarily driven by anxiety or depressive symptoms.^47^ As expected, the general affective symptoms factor was strongly and positively associated with both authoritarian and permissive parenting and negatively correlated with authoritative parenting. The general affective symptoms factor was also associated with punitive and verbally hostile practices, characteristics of authoritarian parenting. These findings align with previous studies linking depressive or anxiety symptoms with parenting.^7^^,^^17^^,^^25^ Notably, permissive and authoritarian parenting were themselves strongly correlated, suggesting that these 2 styles may overlap and reflect broader complexities of parenting. This aligns with previous findings where parents experiencing depressive symptoms or elevated psychological distress may respond with strict discipline at times and be highly lenient at others.^7^ Conversely, the general affective symptoms factor was negatively associated with warmth and supportive authoritative parenting. Additionally, positive mental health was correlated with authoritative parenting and its associated subscales, but not with authoritarian and permissive parenting or their respective subscales, revealing a specific influence on parenting styles.

Permissive and authoritarian parenting mediated the associations between maternal general affective symptoms and childhood total behavioral problems (Table 3). The findings for permissive parenting as mediator also held under stricter inferential criteria, consistent with reports describing the effects of authoritarian or permissive parenting.^48^^,^^49^ Conversely, authoritative parenting mediated the associations between maternal general affective symptoms and poorer cognitive outcomes (Table 3). These outcomes included laboratory and home-based tests of executive function, IQ, and school readiness. Notably, mediation pathways involving child numeracy and receptive verbal competencies as outcomes remained statistically significant even under stricter criteria. Although the total and direct effects of maternal general affective symptoms on school readiness, number knowledge, and executive function tests were not statistically significant, their indirect effects were significant and in opposite directions. The opposing directions of indirect vs total/direct effects suggest possible suppression effects, highlighting the complex interplay between poor maternal mental health and child cognitive outcomes.^50^ These findings underscore the association between negative states of maternal mental health and parenting styles for specific developmental outcomes.

The significance of maternal positive affect in shaping parenting styles that promote optimal developmental outcomes has been underexplored.^51^^,^^52^ Kraybill and Bell^51^ found that maternal positive affect predicted later child executive function. In our study, positive maternal mental health was associated with authoritative parenting, which subsequently mediated the relation between maternal positive mental health and child executive function and cognition (Table 4). This result aligns with our prior findings that positive maternal mental health is associated with cognitive, but not socioemotional, function in infants and toddlers.^17^ The key finding in this study was the notable mediating role of authoritative parenting in the relation between positive maternal mental health and child cognitive development, especially for child receptive vocabulary abilities, where the indirect effect remained robust under a stricter 99% CI BC analysis. Authoritative parenting has been linked to better academic achievement in children.^53^^,^^54^ These findings suggest that positive maternal mental health specifically promotes parenting behaviors associated with optimal cognitive development. The specificity of this pathway is noteworthy as it was not observed with child behavioral problems.

Our study benefits from a wide range of child outcomes, including measures of school readiness and thus likely of importance for academic success. Nevertheless, whereas this report contributes to a broader understanding of maternal mental health and child development, there are a few limitations. We note that our mediation analyses have small effect sizes. However, the significant mediation pathways remain developmentally meaningful given the divergent associations between poor and positive maternal mental health and child outcomes—mediated through varying parenting quality. Importantly, small effects in multiple individual outcomes could accumulate over time and influence a range of developmental domains, especially in early childhood.^55^

Another limitation is the temporal proximity of measurements used in the mediation analyses. Due to the brief, approximately 6-month interval between assessments, the study is cross-sectional in design, hence making it difficult to infer causality of mediation pathways.^56^ Nevertheless, the mediation analyses provide preliminary evidence that parenting may serve as a mechanism linking maternal mental health to child outcomes, highlighting potential pathways for further investigations in longitudinal studies. Our study is also based on maternal-reported measures of well-being and parenting, without incorporating the measures of fathers. Paternal depression is associated with a 42% increased risk of depression in the offspring^57^ and socioemotional problems in young children.^58^ This relation is mediated by influences on family function.^59^ Future studies should indeed focus on positive paternal mental health. We also note that we used maternal reports of parenting and child outcomes at a specific developmental period. Hence, there is a clear need to extend these analyses to include multiple measures of parental care and with children across development.

Our findings reflect a nuanced relation between maternal mental health and parenting. Positive maternal mental health was significantly associated with warmth and reasoning, democratically participative, authoritative parenting. Our findings provide novel evidence for the significance of positive maternal affective states for authoritative parenting, specifically associated with cognitive performance and executive function. These results suggest the importance of public health policies to establish a model of support for mothers to promote positive mental well-being, expanding on the current focus on symptoms of mental disorders. These programs have the potential for impact on both parenting and cognitive outcomes in the future generation.

## CRediT authorship contribution statement

**Michelle Z.L. Kee:** Writing – review & editing, Writing – original draft, Methodology, Investigation, Formal analysis, Data curation, Conceptualization. **Desiree Y. Phua:** Writing – review & editing, Writing – original draft, Visualization, Methodology, Formal analysis. **Helen Y. Chen:** Writing – review & editing, Project administration. **Yap Seng Chong:** Resources, Project administration, Funding acquisition. **Lourdes Mary Daniel:** Writing – review & editing, Resources, Project administration. **Peter D. Gluckman:** Writing – review & editing, Resources, Project administration, Funding acquisition. **Birit F.P. Broekman:** Writing – review & editing, Resources, Project administration. **Michael J. Meaney:** Writing – review & editing, Writing – original draft, Supervision, Resources, Project administration, Conceptualization
