# Supplementary material for: Distinct Roles of Positive and Negative Maternal Mental Health in Parenting Styles and Child Development
Source: JAACAP Open. 2025 Nov 26;4(2):241–53. doi: 10.1016/j.jaacop.2025.11.007 (PMC13043459; doi:10.1016/j.jaacop.2025.11.007)
Supplement: Supplemental Material [file mmc1.pdf]

Table S1: Mean and standard deviations (SD) of measures related to parenting styles and child outcomes

Instruments	Scales	*n*	Mean (SD)
PSDQ	Permissive	328	2.29 (0.63)
	Authoritarian	328	2.25 (0.58)
	Authoritative	328	4.02 (0.53)
Lollipop Test	Total score	300	43.87 (12.77)
Number Knowledge Test	Total score	291	6.26 (3.54)
PPVT	Total score	292	54.99 (20.76)
KBIT2	Composite IQ score	300	94.34 (13.76)
CANTAB - SWM	Total errors (4 to 8 boxes)	300	74.75 (14.89)
CBCL	Total problems	292	30.35 (19.94)

Note: CANTAB – SWM = Cambridge Neuropsychological Test Automated Battery – Spatial Working Memory; CBCL = Childhood Behavioral Checklist (1.5 – 5 years); KBIT2 = Kaufman Brief Intelligence Test version 2; PPVT = Peabody Picture Vocabulary Test (4th edition); PSDQ = Parenting Styles and Dimensions Questionnaire – short version.

Table S1. Comparison of study characteristics between those invited and consented for longitudinal postnatal assessments and those in the current study

	Invited to Study Visit<br>n = 487[a]	Current Study<br>n = 328[a]	*p*-value[b]
Mother's age at delivery, years	31 ± 5	31 ± 5	>.900
Ethnicity			.499
Chinese	270 (55.4)	169 (51.5)	
Malay	136 (27.9)	103 (31.4)	
Indian	81 (16.6)	56 (17.1)	
Maternal highest education level			.030
Secondary and below	152 (31.2)	76 (23.5)	
Pre-tertiary	168 (34.9)	117 (36.1)	
University and above	162 (33.6)	131 (40.4)	
Missing data or refused to answer	5 (1.0)	4 (1.2)	
Monthly household income (SGD)[c]			.070
≤ 1,999	70 (14.4)	33 (10.0)	
2,000 – 3,999	150 (30.8)	89 (27.1)	
4,000 – 5,999	112 (23.0)	79 (24.1)	
> 6,000	127 (26.1)	108 (32.9)	
Missing data or refused to answer	28 (5.7)	19 (5.8)	
Marital status			>.900
Married	465 (95.5)	312 (95.1)	
Single	12 (2.5)	8 (2.4)	
Missing or refused to answer	10 (2.0)	8 (2.4)	
Child sex (Male)	256 (52.6)	168 (51.2)	.760
Child gestational age at delivery, weeks	38.84 ± 1.32	38.86 ± 1.39	.414
Maternal measures			
BDI-II	6.29 ± 7.39	6.24 ± 7.56	>.900
Missing	62 (12.7)	-	
STAI-state	33.90 ± 10.26	33.30 ± 10.58	.432
Missing	63 (12.9)	-	
STAI-trait	36.43 ± 9.67	36.05 ± 10.01	.593
Missing	63 (12.9)	-	

Note:
[a] Mean ± SD; n (%).
[b] Welch Two Sample t-test for continuous variables and Pearson's Chi-squared test for categorical variables.
[c] The acknowledged poverty line is SGD $1,999 monthly income.

Table S3. Exploratory factor analysis model fit statistics

Model	Eigenvalue	Eigenvalue from Parallel Analysis	Model *df*	χ^2	AIC	BIC	Adjusted BIC	RMSEA	RMSEA *p*	CFI	SRMR
1 factor	22.481	1.964	1769	6817.41	33139.49	33833.62	33253.14	0.093	<.001	0.617	0.093
2 factors	4.610	1.879	1709	4897.45	31339.53	32261.23	31490.44	0.075	<.001	0.758	0.061
3 factors	3.030	1.812	1650	4122.05	30682.13	31827.62	30869.69	0.068	<.001	0.812	0.047
4 factors	2.115	1.757	1592	3625.52	30301.60	31667.08	30525.17	0.062	<.001	0.846	0.040
5 factors	1.889	1.707	1535	3134.78	29924.86	31506.55	30183.84	0.056	<.001	0.879	0.033
6 factors	1.330	1.661	1479	2883.38	29785.46	31579.56	30079.22	0.054	.016	0.893	0.030

Note: AIC = Akaike information criteria; BIC = Bayesian information criteria; CFI = Comparison fit index; RMSEA = Root mean square error of approximation; SRMR = Standardized root mean square residual.

Table S4: Factor loadings and contents of items in the Positive Mental Health sub-factor derived from exploratory bi-factor modelling of maternal mental health

**Factor loadings**		**Item**	**Contents of item**
General Affective Symptoms	Factor 2: Positive Mental Health		
-0.48	0.62	STAI Q1	1. I feel calm.
-0.61	0.61	STAI Q15	15. I am relaxed.
-0.61	0.60	STAI Q10	10. I feel comfortable.
-0.53	0.60	STAI Q2	2. I feel secure.
-0.45	0.58	STAI Q5	5. I feel at ease.
-0.64	0.58	STAI Q20	20. I feel pleasant.
-0.55	0.57	STAI Q16	16. I feel content.
-0.65	0.55	STAI Q19	19. I feel steady.
-0.62	0.53	STAI Q8	8. I feel satisfied.
-0.64	0.48	STAI Q11	11. I feel self-confident.
-0.65	0.48	STAI Q33	33. I feel secure.
-0.72	0.46	STAI Q21	21. I feel pleasant.
-0.63	0.46	STAI Q36	36. I am content.
-0.69	0.44	STAI Q30	30. I am happy.
-0.64	0.39	STAI Q27	27. I am calm, cool, and collected.
-0.76	0.36	STAI Q23	23. I feel satisfied with myself.
-0.64	0.32	STAI Q39	39. I am a steady person.

Note: STAI: State-Trait Anxiety Inventory

Table S5: Factor loadings and contents of items in the remaining sub-factors derived from exploratory bi-factor modelling of maternal mental health

**Factor loadings**		**Item**	**Contents of item**
General Affective Symptoms	Factor 3: Sadness		
0.5	0.48	BDI-II Q6	6. Punishment Feelings
0.59	0.46	BDI-II Q1	1. Sadness
0.56	0.46	BDI-II Q8	8. Self-Criticalness
0.58	0.44	BDI-II Q5	5. Guilty Feelings
0.36	0.44	BDI-II Q9	9. Suicidal Thoughts or Wishes
0.56	0.43	BDI-II Q2	2. Pessimism
0.63	0.42	BDI-II Q14	14. Worthlessness
0.66	0.39	BDI-II Q3	3. Past Failure
0.65	0.37	BDI-II Q13	13. Indecisiveness
0.51	0.34	BDI-II Q11	11. Agitation
0.65	0.34	BDI-II Q7	7. Self-Dislike
General Affective Symptoms	Factor 4: Psychosomatic		
0.47	0.67	BDI-II Q20	20. Tiredness or Fatigue
0.52	0.64	BDI-II Q15	15. Loss of Energy
0.35	0.54	BDI-II Q16	16. Changes in Sleeping Pattern
0.56	0.38	BDI-II Q19	19. Concentration Difficulty
0.44	0.36	BDI-II Q21	21. Loss of Interest in Sex
0.55	0.33	BDI-II Q12	12. Loss of Interest
General Affective Symptoms	Factor 5: Anxiety		
0.48	0.52	STAI Q13	13. I am jittery.
0.48	0.51	STAI Q3	3. I am tense.
0.54	0.48	STAI Q12	12. I feel nervous.
0.62	0.4	STAI Q17	17. I am worried.
0.66	0.38	STAI Q6	6. I feel upset.

0.5	0.37	STAI Q9	9. I feel frightened.
0.64	0.31	STAI Q18	18. I feel confused.
0.46	0.3	STAI Q4	4. I feel strained.

Note: BDI-II = Becks’ Depression Inventory (2nd edition); STAI = State-Trait Anxiety Inventory.

Table S6: Reliability indices of the bi-factor confirmatory model of maternal mental health

**Factors**	**Explained Common Variance**	**ω**	**ωh**	**Relative ω**	**H index**	**Factor Determinacy index**
**General**	.74	.95	.65	.68	.98	.99
Factor 2: Positive	.43	.98	.42	.43	.89	.96
Factor 3: Sadness	.16	.97	.15	.15	.60	.85
Factor 4: Psychosomatic	.42	.92	.37	.40	.75	.94
Factor 5: Anxiety	.35	.94	.32	.34	.73	.91

Table S7. Correlations between factor scores of maternal General Affective Symptoms, Positive Mental Health, parenting dimensions, and child outcomes

	1	2	3	4	5	6	7	8	9	10	11	12	13	14	15
1. General Affective Symptoms	-														
2. Positive Mental Health	-0.12 (.034)	-													
3. PSDQ - Indulgent	0.27 (<.001)	0.01 (.831)	-												
4. PSDQ - Warmth and Supportive	-0.16 (.003)	0.14 (.012)	-0.05 (.341)	-											
5. PSDQ – Reasoning / Inductive	-0.16 (.004)	0.23 (<.001)	-0.06 (.270)	0.59 (<.001)	-										
6. PSDQ-Democratic Participative	-0.16 (.004)	0.12 (.027)	-0.02 (.697)	0.62 (< .001)	0.52 (<.001)	-									
7. PSDQ – Physical Coercive	0.20 (<.001)	-0.04 (.431)	0.29 (<.001)	-0.07 (.185)	-0.08 (.134)	-0.09 (.090)	-								
8. PSDQ – Verbally Hostile	0.24 (<.001)	0.04 (.508)	0.42 (<.001)	-0.19 (<.001)	-0.03 (.559)	-0.20 (<.001)	0.51 (<.001)	-							
9. PSDQ – Non-Reasoning/Punitive	0.24 (<.001)	-0.06 (.246)	0.33 (<.001)	-0.09 (.093)	-0.05 (.416)	-0.05 (.321)	0.45 (<.001)	0.45 (<.001)	-						
10. School readiness	-0.002 (.966)	0.09 (.122)	0.09 (.101)	-0.01 (.896)	0.16 (.006)	0.05 (.406)	-0.04 (.464)	0.10 (.083)	-0.06 (.274)	-					
11. Number knowledge	0.03 (.648)	0.08 (.157)	-0.001 (.982)	-0.01 (.858)	0.19 (.001)	0.05 (.409)	-0.07 (.205)	0.07 (.233)	-0.14 (.017)	0.63 (<.001)	-				
12. Receptive vocabulary	-0.10 (.097)	0.15 (.009)	0.07 (.255)	0.04 (.530)	0.20 (<.001)	0.06 (.297)	-0.04 (.532)	0.06 (.303)	-0.08 (.176)	0.63 (<.001)	0.48 (<.001)	-			
13. IQ	-0.12 (.037)	0.20 (.001)	0.01 (.893)	0.06 (.320)	0.16 (.005)	0.05 (.508)	-0.17 (.003)	-0.08 (.159)	-0.16 (.007)	0.52 (<.001)	0.47 (<.001)	0.68 (<.001)	-		
14. Total errors in spatial	-0.003 (.954)	-0.03 (.655)	-0.04 (.462)	-0.06 (.293)	-0.05 (.352)	-0.04 (.476)	0.02 (.677)	-0.02 (.719)	0.07 (.230)	-0.26 (<.001)	-0.24 (<.001)	-0.15 (.017)	-0.19 (.002)	-	

working memory															
15. Total behavioral problems	0.42 (<.001)	0.004 (.941)	0.26 (<.001)	-0.05 (.367)	-0.11 (.056)	-0.11 (.054)	0.18 (.002)	0.24 (<.001)	0.19 (.001)	-0.05 (.371)	-0.05 (.417)	-0.10 (.097)	-0.08 (.222)	-0.02 (.688)	-

Note: Brackets indicate *p*-values; n = 266 – 328.

Table S8. Adjusted and unadjusted mediation effects of parenting styles on the relation between maternal General Affective Symptoms and various child outcomes

Child outcomes	n	Model	Effect Type	Mediator: Authoritarian[a]		Mediator: Authoritative[b]		Mediator: Permissive[a]	
			Effects	ß [95% CI]	ß [99% CI]	ß [95% CI]	ß [99% CI]	ß [95% CI]	ß [99% CI]
School readiness	297	Adjusted	Total	0.016 [-0.095,0.120]	0.016 [-0.131,0.153]	0.014 [-0.097,0.118]	0.014 [-0.135,0.153]	0.017 [-0.094,0.120]	0.017 [-0.131,0.154]
			Direct	0.018 [-0.103,0.129]	0.018 [-0.144,0.168]	0.037 [-0.075,0.147]	0.037 [-0.114,0.176]	0.013 [-0.103,0.121]	0.013 [-0.138,0.160]
			Indirect	-0.001 [-0.034,0.032]	-0.001 [-0.045,0.044]	**-0.023 [-0.057,-0.003]***	-0.023 [-0.072,0.003]	0.004 [-0.029,0.036]	0.004 [-0.042,0.048]
		Unadjusted	Total	-0.002 [-0.115,0.109]	-0.002 [-0.154,0.137]	-0.001 [-0.115,0.11]	-0.001 [-0.155,0.137]	-0.002 [-0.116,0.108]	-0.002 [-0.155,0.136]
			Direct	-0.014 [-0.136,0.105]	-0.014 [-0.175,0.139]	0.021 [-0.100,0.134]	0.021 [-0.140,0.160]	-0.022 [-0.140,0.099]	-0.022 [-0.181,0.131]
			Indirect	0.012 [-0.022,0.050]	0.012 [-0.035,0.062]	**-0.021 [-0.055,-0.001]***	-0.021 [-0.070,0.007]	0.019 [-0.013,0.055]	0.019 [-0.025,0.068]
Number knowledge	288	Adjusted	Total	0.051 [-0.066,0.155]	0.051 [-0.096,0.189]	0.046 [-0.070,0.156]	0.046 [-0.104,0.189]	0.052 [-0.065,0.156]	0.052 [-0.096,0.190]
			Direct	0.078 [-0.043,0.195]	0.078 [-0.074,0.227]	0.070 [-0.047,0.183]	0.070 [-0.083,0.215]	0.061 [-0.065,0.180]	0.061 [-0.100,0.213]
			Indirect	-0.028 [-0.067,0.003]	-0.028 [-0.080,0.013]	**-0.024 [-0.054,-0.004]***	-0.024 [-0.064,0.001]	-0.009 [-0.045,0.025]	-0.009 [-0.060,0.035]
		Unadjusted	Total	0.032 [-0.078,0.142]	0.032 [-0.116,0.171]	0.036 [-0.075,0.148]	0.036 [-0.113,0.177]	0.033 [-0.077,0.144]	0.033 [-0.115,0.172]
			Direct	0.051 [-0.070,0.171]	0.051 [-0.107,0.207]	0.057 [-0.056,0.169]	0.057 [-0.099,0.201]	0.032 [-0.090,0.154]	0.032 [-0.130,0.196]
			Indirect	-0.019 [-0.058,0.015]	-0.019 [-0.070,0.025]	**-0.021 [-0.050,-0.002]***	-0.021 [-0.063,0.004]	0 [-0.037,0.033]	0 [-0.050,0.048]
Receptive vocabulary	289	Adjusted	Total	-0.049 [-0.165,0.069]	-0.049 [-0.206,0.098]	-0.053 [-0.172,0.066]	-0.053 [-0.214,0.098]	-0.049 [-0.164,0.069]	-0.049 [-0.205,0.098]
			Direct	-0.038 [-0.154,0.077]	-0.038 [-0.191,0.112]	-0.025 [-0.144,0.098]	-0.025 [-0.187,0.136]	-0.049 [-0.168,0.072]	-0.049 [-0.203,0.11]
			Indirect	-0.011 [-0.041,0.018]	-0.011 [-0.052,0.030]	**-0.029 [-0.059,-0.009]*****	**-0.029 [-0.072,-0.004]*****	0 [-0.031,0.030]	0 [-0.043,0.043]

		Unadjusted	Total	-0.064 [-0.177,0.061]	-0.064 [-0.207,0.094]	-0.058 [-0.173,0.065]	-0.058 [-0.202,0.098]	-0.065 [-0.179,0.060]	-0.065 [-0.209,0.094]
			Direct	-0.067 [-0.184,0.056]	-0.067 [-0.217,0.094]	-0.032 [-0.152,0.093]	-0.032 [-0.185,0.132]	-0.08 [-0.203,0.050]	-0.08 [-0.237,0.081]
			Indirect	0.004 [-0.027,0.038]	0.004 [-0.039,0.051]	**-0.027 [-0.057,-0.007]*** **	**-0.027 [-0.07,-0.002]*** **	0.015 [-0.014,0.049]	0.015 [-0.024,0.060]
IQ	296	Adjusted	Total	-0.060 [-0.164,0.052]	-0.060 [-0.196,0.086]	-0.059 [-0.168,0.054]	-0.059 [-0.203,0.095]	-0.060 [-0.165,0.051]	-0.060 [-0.197,0.086]
			Direct	-0.023 [-0.135,0.089]	-0.023 [-0.166,0.131]	-0.034 [-0.146,0.083]	-0.034 [-0.183,0.120]	-0.060 [-0.178,0.056]	-0.060 [-0.21,0.093]
			Indirect	**-0.037 [-0.075,-0.008]*** **	**-0.037 [-0.089,-0.001]*** **	**-0.025 [-0.055,-0.005]* **	-0.025 [-0.067,0.001]	0.001 [-0.031,0.030]	0.001 [-0.044,0.040]
		Unadjusted	Total	-0.108 [-0.215,0.005]	-0.108 [-0.246,0.041]	-0.105 [-0.214,0.010]	-0.105 [-0.245,0.046]	-0.107 [-0.214,0.007]	-0.107 [-0.248,0.044]
			Direct	-0.073 [-0.188,0.042]	-0.073 [-0.222,0.086]	-0.087 [-0.200,0.0320]	-0.087 [-0.234,0.066]	-0.113 [-0.227,0.009]	-0.113 [-0.270,0.044]
			Indirect	**-0.034 [-0.075,-0.004]* **	-0.034 [-0.090,0.005]	-0.018 [-0.045,0.002]	-0.018 [-0.057,0.009]	0.006 [-0.023,0.037]	0.006 [-0.035,0.047]
Total errors in spatial working memory[c]	296	Adjusted	Total	0.006 [-0.111,0.121]	0.006 [-0.147,0.155]	0.021 [-0.092,0.127]	0.021 [-0.129,0.164]	0.007 [-0.111,0.12]	0.007 [-0.146,0.156]
			Direct	-0.010 [-0.135,0.109]	-0.010 [-0.172,0.153]	-0.001 [-0.114,0.109]	-0.001 [-0.151,0.145]	0.012 [-0.111,0.132]	0.012 [-0.145,0.176]
			Indirect	0.016 [-0.015,0.053]	0.016 [-0.027,0.065]	**0.022 [0.004,0.052]* **	0.022 [-0.002,0.063]	-0.005 [-0.043,0.027]	-0.005 [-0.057,0.040]
		Unadjusted	Total	0 [-0.116,0.109]	0 [-0.149,0.142]	-0.002 [-0.117,0.106]	-0.002 [-0.151,0.14]	0 [-0.116,0.109]	0 [-0.150,0.142]
			Direct	-0.012 [-0.136,0.107]	-0.012 [-0.172,0.14]	-0.022 [-0.137,0.092]	-0.022 [-0.172,0.129]	0.011 [-0.108,0.125]	0.011 [-0.146,0.163]
			Indirect	0.012 [-0.020,0.048]	0.012 [-0.031,0.062]	**0.020 [0.001,0.05]* **	0.020 [-0.006,0.062]	-0.011 [-0.048,0.02]	-0.011 [-0.062,0.030]
Total behavioral problems[d]	289	Unadjusted	Total	0.442 [0.349,0.527]	0.442 [0.318,0.552]	0.442 [0.352,0.530]	0.442 [0.319,0.556]	0.440 [0.347,0.525]	0.440 [0.315,0.551]
			Direct	0.405 [0.302,0.502]	0.405 [0.266,0.531]	0.437 [0.340,0.527]	0.437 [0.307,0.556]	0.402 [0.301,0.495]	0.402 [0.268,0.522]

			Indirect	**0.037 [0.004,0.083]***	0.037 [-0.006,0.100]	0.006 [-0.014,0.032]	0.006 [-0.023,0.042]	**0.037 [0.010,0.081]****	**0.037 [0.003,0.095]****

Note: ß= Standardized estimates, 95% and 99% CI= Bias-corrected bootstrapping estimation method with a 95% or 99% confidence interval. Bold values indicate significant indirect effects (i.e., 95% or 99% CI do not cross zero).

[a]Adjusted models include maternal highest educational level attained as covariates, except when model includes total behavioral problems as outcome.

[b]Adjusted models include maternal ethnicity and maternal highest educational level attained as covariates, except when model includes total behavioral problems as outcome.

[c]From Cambridge Neuropsychological Test Automated Battery – Spatial Working Memory task.

[d]From Childhood Behavioral Checklist (CBCL). * $p < .05$; ** $p < .01$; *** $p < .005$ after multiple testing corrections.

Table S9. Adjusted and unadjusted mediation effects of parenting styles on the relation between maternal Positive Mental Health and various child outcomes

Child Outcomes	n	Models	Effect Type	Mediator: Authoritarian[a]		Mediator: Authoritative[b]		Mediator: Permissive[a]	
			Effects	ß [95% CI]	ß [99% CI]	ß [95% CI]	ß [99% CI]	ß [95% CI]	ß [99% CI]
School readiness	297	Adjusted	Total	0.068 [-0.033,0.174]	0.068 [-0.068,0.204]	0.066 [-0.033,0.172]	0.066 [-0.072,0.202]	0.068 [-0.031,0.176]	0.068 [-0.067,0.204]
			Direct	0.068 [-0.033,0.175]	0.068 [-0.067,0.203]	0.043 [-0.059,0.153]	0.043 [-0.095,0.191]	0.067 [-0.033,0.174]	0.067 [-0.067,0.204]
			Indirect	0 [-0.006,0.008]	0 [-0.011,0.013]	0.023 [0,0.059]	0.023 [-0.008,0.074]	0.001 [-0.004,0.012]	0.001 [-0.007,0.018]
		Unadjusted	Total	0.086 [-0.024,0.193]	0.086 [-0.060,0.227]	0.082 [-0.028,0.189]	0.082 [-0.062,0.222]	0.085 [-0.026,0.193]	0.085 [-0.061,0.225]
			Direct	0.085 [-0.025,0.193]	0.085 [-0.060,0.228]	0.061 [-0.05,0.172]	0.061 [-0.082,0.202]	0.083 [-0.027,0.191]	0.083 [-0.063,0.225]
			Indirect	0 [-0.005,0.012]	0 [-0.009,0.018]	0.021 [-0.002,0.057]	0.021 [-0.011,0.072]	0.002 [-0.004,0.017]	0.002 [-0.007,0.026]
Number knowledge	288	Adjusted	Total	0.037 [-0.080,0.158]	0.037 [-0.115,0.191]	0.040 [-0.074,0.156]	0.040 [-0.104,0.191]	0.037 [-0.08,0.157]	0.037 [-0.113,0.191]
			Direct	0.039 [-0.078,0.158]	0.039 [-0.111,0.189]	0.017 [-0.099,0.136]	0.017 [-0.130,0.172]	0.037 [-0.080,0.158]	0.037 [-0.113,0.192]
			Indirect	-0.002 [-0.019,0.006]	-0.002 [-0.027,0.010]	**0.023 [0.001,0.057]***	0.023 [-0.005,0.068]	-0.001 [-0.012,0.004]	-0.001 [-0.018,0.007]
		Unadjusted	Total	0.061 [-0.056,0.178]	0.061 [-0.099,0.216]	0.059 [-0.058,0.175]	0.059 [-0.101,0.214]	0.060 [-0.057,0.177]	0.060 [-0.098,0.214]
			Direct	0.061 [-0.057,0.175]	0.061 [-0.097,0.213]	0.021 [-0.002,0.052]	0.021 [-0.010,0.064]	0.060 [-0.057,0.176]	0.060 [-0.097,0.215]
			Indirect	-0.001 [-0.015,0.005]	-0.001 [-0.022,0.010]	0.038 [-0.082,0.159]	0.038 [-0.120,0.197]	0 [-0.006,0.010]	0 [-0.010,0.015]
Receptive vocabulary	289	Adjusted	Total	0.094 [-0.019,0.209]	0.094 [-0.051,0.247]	0.103 [-0.009,0.215]	0.103 [-0.046,0.252]	0.095 [-0.019,0.209]	0.095 [-0.05,0.247]
			Direct	0.095 [-0.020,0.209]	0.095 [-0.050,0.253]	0.072 [-0.042,0.185]	0.072 [-0.074,0.220]	0.095 [-0.020,0.211]	0.095 [-0.051,0.249]
			Indirect	-0.001 [-0.015,0.005]	-0.001 [-0.023,0.008]	**0.031 [0.009,0.066]*****	**0.031 [0.004,0.081]*****	-0.001 [-0.013,0.003]	-0.001 [-0.019,0.006]

		Unadjusted	Total	0.115 [-0.003,0.235]	0.115 [-0.039,0.273]	0.113 [-0.004,0.233]	0.113 [-0.040,0.273]	0.115 [-0.002,0.234]	0.115 [-0.039,0.273]
			Direct	0.115 [-0.004,0.234]	0.115 [-0.040,0.276]	0.084 [-0.033,0.209]	0.084 [-0.074,0.251]	0.114 [-0.004,0.232]	0.114 [-0.041,0.270]
			Indirect	0 [-0.008,0.005]	0 [-0.013,0.01]	**0.029 [0.008,0.062]*****	**0.029 [0.001,0.076]*****	0.001 [-0.003,0.014]	0.001 [-0.006,0.022]
IQ	296		Total	0.134 [0.024,0.244]	0.134 [-0.009,0.276]	0.132 [0.023,0.240]	0.132 [-0.005,0.274]	0.131 [0.022,0.242]	0.131 [-0.011,0.275]
			Direct	0.137 [0.026,0.246]	0.137 [-0.006,0.280]	0.107 [-0.007,0.229]	0.107 [-0.040,0.266]	0.132 [0.022,0.243]	0.132 [-0.010,0.275]
			Indirect	-0.003 [-0.022,0.013]	-0.003 [-0.029,0.019]	**0.025 [0.003,0.064]***	0.025 [-0.005,0.081]	-0.001 [-0.012,0.003]	-0.001 [-0.015,0.006]
		Unadjusted	Total	0.151 [0.038,0.262]	0.151 [-0.004,0.295]	0.148 [0.035,0.261]	0.148 [-0.005,0.295]	0.148 [0.036,0.261]	0.148 [-0.005,0.293]
			Direct	0.152 [0.038,0.263]	0.152 [0.003,0.297]	0.129 [0.006,0.251]	0.129 [-0.030,0.290]	0.149 [0.036,0.261]	0.149 [-0.003,0.295]
			Indirect	-0.002 [-0.020,0.015]	-0.002 [-0.028,0.022]	0.019 [-0.004,0.053]	0.019 [-0.014,0.069]	0 [-0.011,0.003]	0 [-0.016,0.006]
Total errors in spatial working memory[c]	296	Adjusted	Total	-0.019 [-0.129,0.094]	-0.019 [-0.160,0.124]	-0.028 [-0.135,0.084]	-0.028 [-0.164,0.117]	-0.018 [-0.128,0.094]	-0.018 [-0.160,0.123]
			Direct	-0.021 [-0.130,0.092]	-0.021 [-0.163,0.123]	-0.002 [-0.112,0.113]	-0.002 [-0.150,0.144]	-0.018 [-0.127,0.096]	-0.018 [-0.160,0.128]
			Indirect	0.001 [-0.005,0.016]	0.001 [-0.009,0.022]	**-0.026 [-0.061,-0.003]****	-0.026 [-0.075,0.005]	-0.001 [-0.012,0.004]	-0.001 [-0.017,0.008]
		Unadjusted	Total	-0.015 [-0.120,0.092]	-0.015 [-0.153,0.123]	-0.012 [-0.118,0.095]	-0.012 [-0.151,0.124]	-0.013 [-0.120,0.094]	-0.013 [-0.152,0.125]
			Direct	-0.015 [-0.121,0.092]	-0.015 [-0.153,0.122]	0.012 [-0.099,0.125]	0.012 [-0.134,0.161]	-0.012 [-0.120,0.095]	-0.012 [-0.152,0.125]
			Indirect	0 [-0.005,0.012]	0 [-0.009,0.018]	-0.024 [-0.058,0.001]	-0.024 [-0.072,0.009]	-0.001 [-0.016,0.003]	-0.001 [-0.024,0.006]
Total behavioral problems[d]	289	Unadjusted	Total	-0.048 [-0.165,0.072]	-0.048 [-0.196,0.106]	-0.049 [-0.166,0.071]	-0.049 [-0.199,0.105]	-0.044 [-0.159,0.076]	-0.044 [-0.193,0.112]
			Direct	-0.051 [-0.167,0.067]	-0.051 [-0.201,0.100]	-0.026 [-0.143,0.097]	-0.026 [-0.173,0.135]	-0.051 [-0.162,0.062]	-0.051 [-0.199,0.097]

			Indirect	0.003 [-0.026,0.032]	0.003 [-0.038,0.042]	-0.023 [-0.063,0.001]	-0.023 [-0.076,0.010]	0.008 [-0.017,0.037]	0.008 [-0.029,0.048]

Note: ß= Standardized estimates, 95% and 99% CI= Bias-corrected bootstrapping estimation method with a 95% or 99% confidence interval. Bold values indicate significant indirect effects (i.e., 95% or 99% CI do not cross zero).

[a]Adjusted models include maternal highest educational level attained as covariates, except when model includes total behavioral problems as outcome.

[b]Adjusted models include maternal ethnicity and maternal highest educational level attained as covariates, except when model includes total behavioral problems as outcome.

[c]From Cambridge Neuropsychological Test Automated Battery – Spatial Working Memory task.

[d]From Childhood Behavioral Checklist (CBCL). * $p < .05$; ** $p < .01$; *** $p < .005$ after multiple testing corrections.
